# Disentangling concepts of inappropriate polypharmacy in old age: a scoping review

**DOI:** 10.1186/s12889-023-15013-2

**Published:** 2023-02-04

**Authors:** Sofie Rosenlund Lau, Frans Waldorff, Anne Holm, Anne Frølich, John Sahl Andersen, Mette Sallerup, Sarah Emilie Christensen, Stine Søndersted Clausen, Tina Drud Due, Pernille Hølmkjær

**Affiliations:** 1 The Research Unit for General Practice and Section of General Practice, Department of Public Health, University of Copenhagen, Copenhagen, Denmark; 2 Innovation and Research Centre for Multimorbidity, Slagelse Hospital, Slagelse, Denmark

**Keywords:** Polypharmacy, Older adults, Geriatrics, Conceptualization, Standardization, Practices, Politics

## Abstract

**Introduction:**

Polypharmacy is a common concern, especially in the older population. In some countries more that 50% of all individuals over 60 receive five or more drugs, most often due to multimorbidity and increased longevity. However, polypharmacy is associated with multiple adverse events, and more medication may not always be the answer. The terms “appropriate” and “inappropriate” are often used to distinguish between “much” and “too much” medications in relation to polypharmacy in research and practice, but no explicit definition exists to describe what these terms encompass. The aim of this review is to unfold the different understandings of and perspectives on (in)appropriate polypharmacy and suggest a framework for further research and practice.

**Method:**

A scoping review was conducted using the framework of Arksey and O’Malley and Levac et al. Pubmed, Embase, PsycINFO, CINAHL, Cochrane database, Scopus and Web of Science were searched for references in English, Danish, Norwegian and Swedish using the search string “Polypharmacy” AND “Appropriate” OR “Inappropriate”. Data was extracted on author information, aims and objectives, methodology, study population and setting, country of origin, main findings and implications, and all text including the words “appropriate,” “inappropriate,” and “polypharmacy.” Qualitative meaning condensation analysis was used and data charted using descriptive and thematic analysis.

**Results:**

Of 3982 references, a total of 92 references were included in the review. Most references were from 2016-2021, from fields related to medicine or pharmacy, and occurred within primary and secondary healthcare settings. Based on the qualitative analysis, a framework were assembled consisting of *Context*, three domains (S*tandardization, Practices* and *Values & Concerns*) and *Patient Perspective*.

**Conclusion:**

Inappropriate polypharmacy is a concept loaded by its heterogeneity and the usefulness of a single definition is doubtful. Instead, the framework suggested in this article representing different dimensions of inappropriate polypharmacy may serve as an initial strategy for focusing research and practice on polypharmacy in old age.

**Supplementary Information:**

The online version contains supplementary material available at 10.1186/s12889-023-15013-2.

## Introduction

Polypharmacy is a global concern on the rise, most common in the older population [1]. Polypharmacy is most commonly understood to indicate the use of five or more drugs. A Danish study found that around 53% of all persons older than 60 years use medications from more than five different drug classes [2]. A similar high prevalence of multiple medication usage has been detected in various other countries [3, 4].

Polypharmacy is often seen as a consequence of increased longevity and the subsequent occurrence of multiple chronic conditions, and therefore often deemed necessary in order to address symptoms and prevent complications of multimorbidity [5]. However, the use of multiple medications may also cause harm. Studies have shown that polypharmacy increases the risk of adverse outcomes, including mortality, falls, adverse drug reactions, and increased hospitalization and readmissions [6–11]. As such, polypharmacy may affect both the health and wellbeing of individuals and represent a burden on healthcare systems.

Both academia and clinical practice continue to discuss the definition of polypharmacy, when polypharmacy becomes problematic, and the effects of polypharmacy on health and wellbeing [12–14]. Consensus seems to agree that the factors driving polypharmacy are many and arise from multiple issues. Swinglehurst and Fudge denote polypharmacy as a wicked problem “comprising a complex tangle of the biological, behavioral, technological, cultural, and sociopolitical” (p. 389) with no amenable “quick fix” [15, 16]. Similarly, there seems to be a consensus that polypharmacy is not necessary problematic per se: terms like *appropriate* and *inappropriate polypharmacy* are commonly used, especially in the scientific literature, to highlight this distinction between “many” and “too many” medications [13, 14]. However, no consensus yet exists as to how to define and differentiate various dimensions of appropriate and inappropriate polypharmacy, which appears in the literature as a heterogeneous and unclear concept [12, 14, 16]. This heterogeneity in definitions and understandings of what constitutes problematic polypharmacy constitutes a huge challenge for both research and practice, as it makes it difficult to determine and understand consequences related to polypharmacy or to suggest new ways to improve practice [14].

To gain an understanding of the complexity of polypharmacy, Armstrong and Swinglehurst call for an unpacking of the terms “appropriate” and “inappropriate” [12]. In order to do this, we conducted a scoping review following the recommendations of Arksey and O’Malley as well as that of Levac et al. [17, 18] No universal definition exists for scoping reviews, but overall the definitions commonly refer to rapidly map the key concepts of a research area [18, 19]. The scoping review is a relevant method when attempting to identify gaps in a vast amount of literature and requires analytical reinterpretation [18]. A systematic review is a summary of the medical literature that uses explicit and reproducible methods to systematically search, critically appraise, and synthesize on a specific issue [20]. Since we aim to contribute with answers to the call of unpacking the terms by exploring conceptualizations of inappropriate polypharmacy in the literature, the scoping review following the framework provided by Arksey and O’Malley is a well used method [21–23]. We specifically explore (i) conceptualizations of “inappropriateness” in relation to polypharmacy in old age in scientific references, (ii) in what context, including scientific fields, these references are conducted, and (iii) how they involve aspects of the patient perspective. The disentanglement of this complex phenomenon is important as a way to move forward in the search for solutions and to reduce risks associated with polypharmacy, both in research and in practice.

## Methods and analysis

### Design

This scoping review was conducted using the scoping review methodological framework described by Arksey and O’Malley together with the recommendations of Levac et al. [17, 18]. The original framework by Arksey and O’Malley includes the following five key stages: identifying the research question, identifying relevant studies, study selection, charting the data and collating, and summarizing and reporting the results. With the recommendations of Levac et al each stage was enhanced to further improve the framework. This scoping review was developed and carried out within a research team consisting of physicians, anthropologists, pharmacologists and masters of public health science. The main authors were two physicians and a pharmacologist. Throughout the process, team meetings were held and each step was discussed, from generating the idea to finalizing the article. Based on discussions in our polypharmacy research team, we identified a potential gap in the definition of “inappropriate polypharmacy.” The research question was then reformulated and further defined through an iterative process involving all members of the research team. The scoping review is reported according to the PRISMA extension for Scoping Reviews [24].

### Eligibility criteria

Informed by the research question, eligible papers were identified by consensus among the research team members. Included studies needed to have a primary focus on polypharmacy and the terms appropriate or inappropriate had to be an essential part. Furthermore, the studies had to be concerned with multimorbidity or co-morbidity for persons older than 65. There was no limitation on year of publishing or type of studies. Title and abstract had to be available and studies needed to be in English, Danish, Swedish or Norwegian. Studies were excluded if they were outside the scope of the research question, e.g., animal studies, those focused on a single disease, or those with a primary focus on acute illness. These criteria were tested on a sample of abstracts to determine whether they captured studies with the potential to answer the research question.

### Information sources

In order to identify and include relevant papers, an information specialist at the University of Copenhagen was consulted. Based on the broad perspective of our research question, we agreed upon a simple search string including “Polypharmacy” AND “Appropriate” OR “Inappropriate”. A random subsample of references from the preliminary search was assessed and no additional search terms were identified. To ensure as broad a coverage as possible, the following databases were included: Pubmed, Embase, PsycINFO, CINAHL, Cochrane, Scopus and Web of Science. The database search was performed between the 24^th^ of November 2020 and 7^th^ of December 2020. Additionally, we contacted research collaborators in Scotland and Norway in order to identify potential additional references not published in the indexed databases, but no additional references were identified. An updated search on Pubmed was performed on the 24^th^ of October 2022.

### Search Strategy

The search strategy is presented in Table 1.

**Table 1:** The results of the search strings

**Database and date**	**Search strings**	**Hits**
Pubmed 24/11-2020 *Updated search (year 2021-2022)* *Pubmed 24/10 2022*	((appropriate*) OR (inappropriate*)) AND ((polypharmac*) OR ("Polypharmacy"[Mesh]))	2281 *630*
Embase 1/12-2020	((appropriate*) OR (inappropriate*)) AND ((polypharmac*) OR ("Polypharmacy"[Mesh])) Abstract status papers excluded	2824
CINAHL 24/11-2020	( TITLE-ABS-KEY ( polypharmac* ) ) AND ( appropriate* OR inappropriate* )	872
PsycINFO 24/11-2020	( TITLE-ABS-KEY ( polypharmac* ) ) AND ( appropriate* OR inappropriate* )	305
Scopus 7/12-2020	( TITLE-ABS-KEY ( polypharmac* ) ) AND ( appropriate* OR inappropriate* )	5653
Web of science 1/12-2020	(polypharmacy*) AND (appropriate* OR inappropriate*)	2107

### Selection of source of evidence

The results obtained from each search were downloaded into Endnote, where duplicate records were removed. The remaining results were then imported into Covidence and if any duplicates were still present, they were removed using the automatic duplicate removal tool. A two-stage study selection process comprising of [1] screening of titles and abstracts, and [2] a full-text screening including data extraction was conducted.

Following the recommendations made by Levac et al. [18], two reviewers (SRL and PH) independently inspected titles and abstracts to identify potentially relevant studies. All references considered relevant by either reviewer were included for full-text evaluation. If the full text was unavailable, the reference was excluded. Any disagreements in the selection process were resolved through discussion with a third reviewer (FW).

The full-text screening was done in collaboration with the entire research team and was based on a reading template which also comprised a data extraction template (described in detail below).

Quality assessment is not an integral part of this type of scoping review since the aim is to clarify concepts and not provide an overview of the literature or identify gaps in the available evidence [25].

### Data charting and extraction

For the full-text review and extraction of data, a reading- and data extraction template was developed in order to extract data systematically. The template was pilot-tested on 10 references by the research team in April 2021. Based on the pilot testing, the template was adjusted and thereafter used for full-text screening and data extraction (Supplementary material 1). All available full-text references were assessed independently by two team members using the template. The two extraction templates were then merged and redundant material was deleted in consensus with an independent researcher who was not part of the data extraction team. All unique templates were evaluated by SRL and PH independently. Based on the templates, references that did not meet the inclusion criteria at this stage were excluded. Any disagreements were discussed and resolved between SLR, PH and FW.

### Data items

Based on the template, data was extracted on author information (title, author, year of publication, DOI), aims and objectives, methodology used (e.g., quantitative, qualitative, etc.), study population, setting (when relevant), country of origin, main findings and implications, and all text parts including the word “appropriate” (which also includes “inappropriate”) and “polypharmacy.”

The text parts concerning the words appropriate, inappropriate and polypharmacy were identified using the search tool in Adobe Acrobat. Furthermore, the research team could include a comment on any given reference if they identified important information that was not included in the template (e.g., if it should be excluded).

### Synthesis of results

We followed the standards of a classical qualitative meaning condensation analysis, moving from a total, unstructured impression of the data to sorting the data under specific codes and themes and finally synthesizing these themes while suggesting new ways of interpreting meanings and concepts [26, 27].

A twofold analytical strategy, consisting of a descriptive analysis and a thematic analysis, was used to synthesize the charted data. First all the includes references and the reading templates were imported into QSR International’s qualitative data analysis software NVivo®, which was used to facilitate the coding.The descriptive analysis included the following codes: the year of publication, setting, scientific field, geographical location, and methodology for each of the included references. These elements were primarily drawn from the templates. In cases of doubt, the full texts were reassessed. Scientific field was assessed based on the journal of publication. If the journal title did not provide enough information, the journal site was searched for accepted research areas.

For the thematic analysis, a qualitative inductive approach was used, inspired by Malterud’s systematic text condensation, which aims to help researchers organize and interpret qualitative data material [26]. One researcher (SRL) read the full dataset without coding to get an overall impression of the results. Subsequently, SRL suggested an initial set of codes relating to 1) three different ways of interpreting the understandings of inappropriate polypharmacy (initially standards, practices and politics), and 2) the inclusion of patient perspectives.

This approached was discussed within the research team. Finally, the analysis was presented and discussed amongst all authors during a three-hour online workshop. After this workshop, SRL recoded all the material again using NVivo®. Afterwards, the software was used to sum-up and count all the codes from the descriptive analysis and to get an overview of the different domains in the thematic analysis. To present the results of the analysis, a conceptual figure was also developed.

## Results

### Results of the search

**Fig 1:**
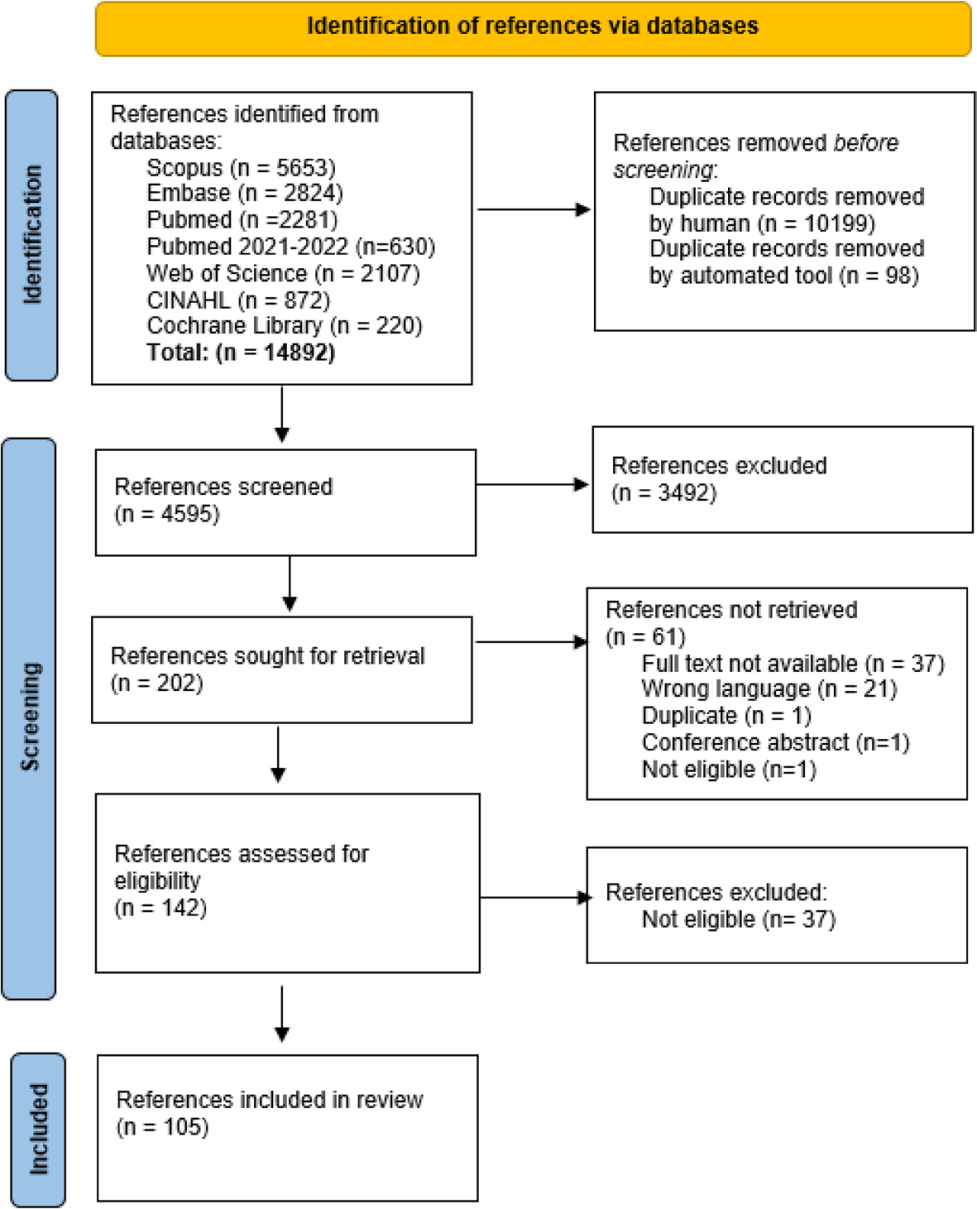
Flow chart of study selection

The complete flowchart of the search is presented in Fig. 1. After the removal of duplicates, a total of 3982 title/abstracts were screened. Of these, 3807 were not eligible according to the criteria previously discussed. This resulted in 175 references eligible for full text screening. Of these, 122 references were available for the final full text screening and data extraction by the research team. Based on the full text reading, 30 references were excluded, and a total of 92 references were included in the review. The additional search performed in 2022 resulted in 613 new papers after duplications were removed. Of these, 19 were eligible for full text screening. This resulted in 13 references to be included in the final analysis. A total of 105 references were included in the final review. No additional gray literature was obtained.

In the following, we present the findings from the qualitative analysis. First, we describe the context of the conceptualizations of inappropriate polypharmacy based on the descriptive analysis of the results. Then, based on the thematic analysis, we present three domains, each representing one way of interpreting the understandings of inappropriate polypharmacy. Finally, we elaborate on aspects of patient perspectives found in the reviews.

### Context

The conceptualizations of inappropriate polypharmacy in the references examined can be understood as situated within a series of surrounding factors in the following referred to as *Context* (see Table 2; see additional details in Appendix 2). The majority of the references are from 2016 through 2020, with an equal distribution of studies conducted in primary and secondary healthcare settings. Most references were published in journals or books related to medicine or other scientific fields within health sciences, e.g., pharmacy, nursing or public health. Only three references were published elsewhere: one in a dissertation abstract journal, one in *Systematic Reviews* and one as a lecture note in engineering and computer sciences. The vast majority originate from Western countries, especially Europe and the US. Finally, we found an even distribution of methodologies within the reference sample, with many commentaries or discussion papers.

**Table 2:** Overview of contextual factors for the articles concerning polypharmacy and inappropriateness

Total studies included: 105			
**Year of publication**	**Years**	**Number**	**Percentage**
	1990-1995	1	1%
	1996-2000	3	3%
	2001-2005	6	6%
	2006-2010	17	16%
	2011-2015	11	10%
	2016-2020	54	51%
	2021-2022	13	12%
	Total	105	100%
**Setting**	**Setting**	**Number**	**Percentage**
	Hospital	20	19%
	Primary care including nursing homes	28	27%
	Cross-sectional	20	19%
	Not applicable	37	35%
	Total	105	100%
**Scientific field**	**Field**	**Number**	**Percentage**
	Medicine	61	58%
	Other fields within the health sciences	41	39%
	Other fields not primarily related to health sciences	3	3%
	Total	105	100%
**Geographical place**	**Place**	**Number**	**Percentage**
	Scandinavia	7	7%
	Europe	53	50%
	USA/Canada	27	26%
	Asia	7	7%
	South and Central America	1	1%
	Australia and New Zealand	8	8%
	Sub-Saharan Africa	2	2%
	Total	105	100%
**Methodology**	**Design**	**Number**	**Percentage**
	Qualitative	16	15%
	Quantitative	21	20%
	Mixed-methods	3	3%
	Reviews (Including systematic reviews)	33	31%
	Commentaries/discussion papers/editorials	28	27%
	Book chapters	4	4%
	Total	105	100%

### Domains

**Fig 2:**
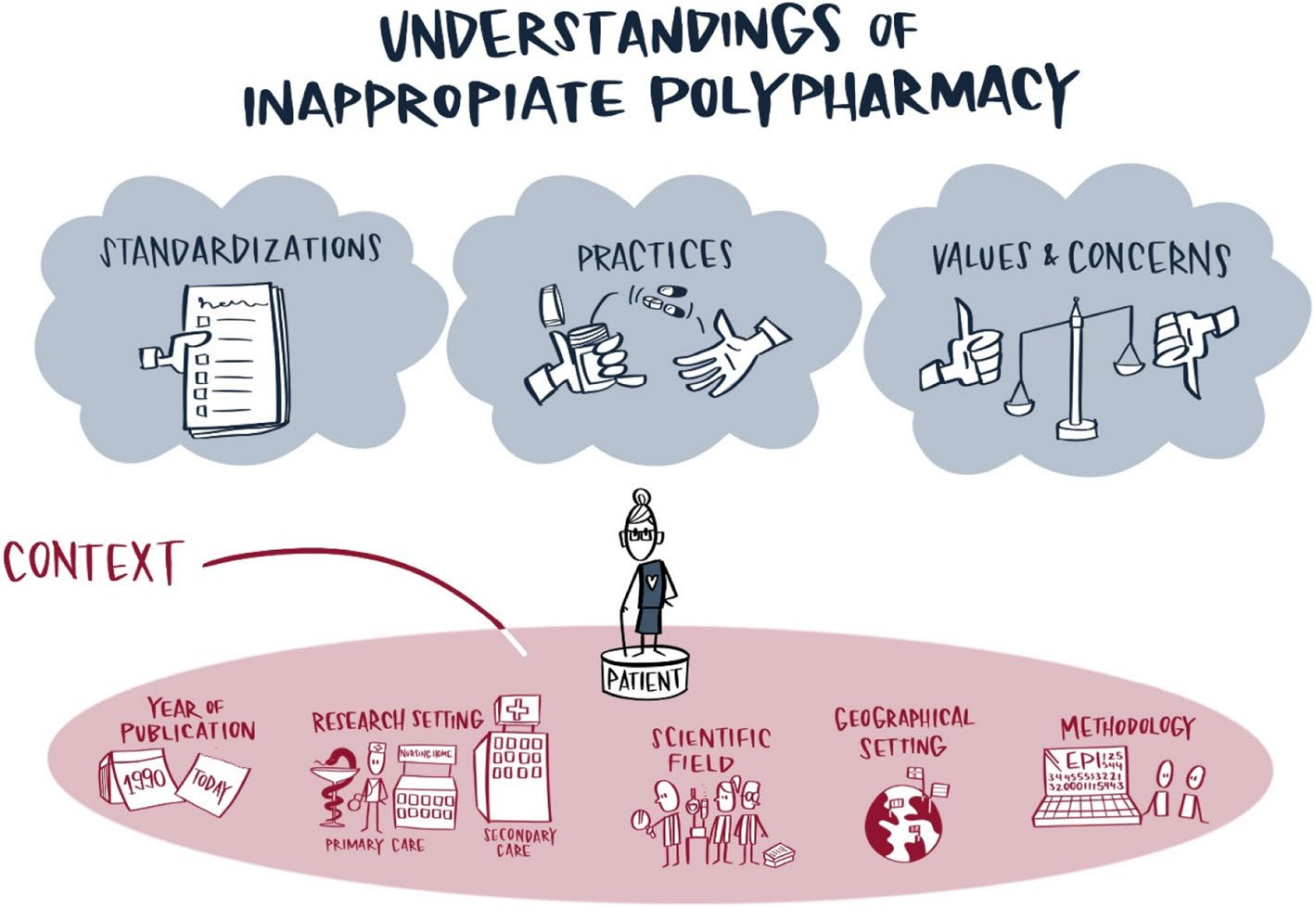
The figure illustrates the condensation of understandings of (in)appropriate polypharmacy found in the reviewed literature. These different domains, the context in which they are placed, and their relation to individual patients represent the heterogeneity of conceptualizations of inappropriate polypharmacy, and thus are important to address in future studies related to the potential problems arising in the wake of polypharmacy. Illustration by Kirstine Kolling, ©Tusamotus used under the Creative Commons Attribution 4.0 International Licence

Based on the thematic analysis, we identified three different ways of characterizing approaches to inappropriate polypharmacy: (i) as a standardized way of interpreting inappropriate polypharmacy; (ii) as a social practice involving different people in different settings; and (iii) from a broader, more political perspective of values and concerns. In the following, these are referred to as the three domains of S*tandardization, Practices* and *Values & Concern*s. This is not to indicate that each reference explicitly covers a specific domain per se, but rather to provide a general reading of these domains across all included references. Therefore, each reference may refer to several domains. The inter-relation between the three domains, the context and the patient perspective is presented in Fig. 2.

#### Standardization

The first domain refers to inappropriate polypharmacy mainly as a set of specific measures, norms or models, which we here summarize under the common term *Standardization*. By standardization, we mean a specific principle or level of quality which is often used as a comparative measure. Examples of different standardization tools include the Systematic Tool to Reduce Inappropriate Prescribing (STRIP), The European STOPP/START 2015, American Geriatric Society 2015 Beers Criteria, or more or less validated lists of potentially inappropriate medications (PIMs) [28, 29]. Some of these tools are developed to target older persons with polypharmacy, but mostly they evaluate if there is inappropriate medication under a specific condition with a single drug. They are almost exclusive used for patients with polypharmacy and may therefore be a proxy for describing inappropriate polypharmacy. By using the lists, reductions or additions of specific medication will add to improve the appropriateness of polypharmacy.

50 of the reviewed references include some elements of standardized notions of inappropriate medication for a population of patients with polypharmacy. Studies using these standards often provide information on the prevalence of inappropriate polypharmacy in a specific population or propose possibilities for adjusting the standardization to specific settings. For instance, Matanović and Vlahović-Palčevski assess a newly developed screening tool for detecting potentially inappropriate medications by comparing it with the 2012 Beers Criteria in a population of hospitalized patients [30]. Stuijt et al. assess the reliability of the Medication Appropriateness Index score in a Dutch nursing home [31]. Advinha et al. assess the complexity of medication regimens in institutionalized older adults using the medication regime complexity index (MRCI) [32].

### Practices

The second domain, *Practices,* refers to inappropriate polypharmacy as a practice taking place in a “real-world setting” between individuals, e.g. healthcare professionals and/or patients and relatives, and is included in 36 of the reviewed references. This approach differs from the above-mentioned standardization approach by relating inappropriate polypharmacy to the dynamic, often more complex aspects of clinical practice and everyday care of polypharmacy patients. The research studies using this approach are concerned with the practical management of medications, including clinical decision-making, prescribing, retrieving, dosing, giving and taking medications. Thus, this domain often considers medication practices from the perspective of different healthcare professionals and in specific care settings. For instance, Christensen et al., while referring to other research, found that the working environment of physicians was counterproductive to appropriate prescribing due to heavy workloads, interruptions while prescribing and the lack of supportive IT systems [15]. Gabauer et al. argue that nurses can play an important role in explaining care goals to patients, including encouraging the deprescribing of inappropriate medication [33].

This domain also includes understandings of inappropriate prescribing as a process that occurs over time. Morgan states that certain medications can be appropriate at the time of prescribing, but the patient's situation might change, making the prescription inappropriate if the medication is not reviewed continuously [34]. Morin et al. argue that “good prescribing is not only about knowing when to start and when to stop treating patients, but also about knowing when not to start in the first place” [35].

Frequent and systematic medication reviews are often suggested as the solution to inappropriate polypharmacy within this domain. However, the question remains: by whom, and when should these reviews be undertaken? While many suggest that the general practitioner plays an important role, other health care professionals especially pharmacists and nurses, are also mentioned as potential important stakeholders in the management of inappropriate polypharmacy. At the same time however, many studies also expressed concern about the risks associated with fragmented care provided by multiple professionals.

### Values & Concerns

The final domain explores inappropriate polypharmacy from the broader perspective of *Values & Concerns*, as found in 49 of the reviewed references. Studies in this domain often take as the point of departure an ideological or political approach to medication use without referring to specific settings or defined practices in which polypharmacy occurs. Definitions in this domain refer to polypharmacy as a balancing act with both positive and negative sides, often using quite vague jargon with no clear cuts or definitions. The naming of the domain thus refers to the way many of the references allude to polypharmacy as simultaneously “good” and “bad,” and the complexity arising from this ambiguity. For instance, Bennett et al. describe inappropriate or problematic polypharmacy as “inappropriate prescribing where the potential harms of individual medicines, prescribed in combination with several other medicines, outweigh the benefits” [36]. Kaufman, while referring to other research, states that “What constitutes ‘many’ or ‘too many’ is a dilemma for prescribing practitioners, and choosing the best treatments that aim to ensure appropriate polypharmacy is a challenge for healthcare professionals and organisations” [37]. Similarly, Medeiros-Souza refers to appropriate prescribing as “balanced prescription” which considers the physiological changes of older adults and the adverse effects of the drugs [38].

In this domain, special attention is given to more political notions behind the current incidences of potentially inappropriate polypharmacy. For instance, McIntosh et al. mention the exclusion of older adults from clinical trials, lack of focus in clinical practice guidelines on issues such as screening and prevention in older patients, and lack of consensus on the treatment of advanced disease near the end of life as important aspects of inappropriate polypharmacy [39]. This domain therefore also includes an economic perspective, as seen in Rocchiccioli et al., who argue that the full cost to the US health care system for inappropriate use of medication may be more than $100 billion annually [40].

Solutions within this domain are often more complex than changing individual practices (e.g., introducing a standardized tool to improve prescribing routines) and take a more holistic view of the problem, including the lack of evidence, educational aspects and the need for improving entire healthcare systems. For example, Gabauer et al. suggest that a multidisciplinary, systematic approach is needed, including institutional policies and financial incentives which align with the process of appropriate polypharmacy throughout the entire US health care system [33]. McGavock calls for urgent action by health administration, epidemiologists, medical educators and prescribing doctors [41].

### Patient perspective

In general, we found that the majority of references communicate to and from the perspective of healthcare professionals, either from/to the position of the same professional, e.g. pharmacists, nurses or medical doctors, or from one healthcare professional to another, e.g. pharmacists to medical doctors. Only one reference, Sperling et al., explicitly states that the overall aim of the study is to help the consumer make a more informed decision about medications [42]. Other references mention that patients and healthcare professionals might examine inappropriate polypharmacy from different perspectives or rationales. For instance, Brahma, Marak and Wahlang argue that the rational use of medicines can be understood from both a medical therapeutic view and a consumer perspective. The consumer perspective is based on the medicines’ value in daily life and can be influenced by cultural perceptions and economic conditions [43]. However, the majority of references do not include patient perspectives in their studies or discussions of the concept of inappropriate polypharmacy.

## Discussion

There is an extensive body of literature addressing inappropriate polypharmacy but a lack of consensus on its core dimensions. Based on this scoping review, we suggest the three domains of *Standardizations*, *Practices*, and *Values & Concerns* as a template for future studies on inappropriate polypharmacy.

Within the *Standardization* domain, many of the assessment tools explained in the references represent different approaches to solving the challenges of inappropriate polypharmacy, often in the form of decision support tools or clinical guidelines which can be used to assess the quality or “rightness” of already prescribed medications. This domain is commonly represented within prevalence studies to provide an overview of the amount of inappropriate polypharmacy within a population or in trials to reduce polypharmacy [44, 45]. *Standardization* is necessary in such situations to apply a certain level of quality and rigor to the studies. At the same time, due to the many possible uses and populations, the standardized approach risk to represent polypharmacy as a rather generic problem, while instead it must be adapted to the specific settings in which such tools are to be used. For instance, standardization tools made for older adults at the end of life may not be relevant for non-terminal persons [46, 47]. Therefore, when examining inappropriate polypharmacy from the perspective of standardization, it becomes extremely important to differentiate between different patient subpopulations with different treatment goals and potential preferences in relation to the outcome of their pharmacological treatment.

The domain of *Practices* moves the understanding of polypharmacy away from standardized notions of tools and guidelines and towards perceiving inappropriate polypharmacy as something that happens among and between different people and professions. It thus moves the focus on solutions primarily concerned with medication lists to broader organizational aspects of the prescribing situation, e.g. the often busy working environment or the sharing of tasks and responsibilities across different professions (e.g. physicians, nurses and pharmacists) [48–51]. While solutions are definitely becoming more complex within this domain, it simultaneously holds the potential for better implementation of e.g. decision tools, as it includes a critical perspective on the structural context of prescribing and medication management.

The final domain, *Values & Concerns*, moves even further away from individual medication lists to understanding inappropriate polypharmacy from a systems approach, which includes also the lack of scientific evidence and the structuring of entire healthcare systems. While the understanding of inappropriate polypharmacy within this domain tends to be too generalized and lacks substantial meaning (e.g., “a balancing act”), it also has potential to broaden the field by exploring fundamental, underlying and holistic aspects and solutions to the problems caused by and arising from the prescription of too much medication in older adults.

An important factor to include when discussing polypharmacy is the patient perspective, especially given the recent increase in involving patients in their own treatment [52–54]. While this trend was not observed to be dominant in this scoping review, we find it important to discuss how the patient perspective was represented in each of the three domains. First, the domain of *Standardizations* includes a great variety of methods of standardization, and hence a great diversity in the extent of patient perspectives included across the included references. While some did not mention patient perspective at all, others were more explicitly patient-centered, e.g. with a tool that directly asked the patient “What matters to you,” or with discussions of why both the health care professional and the patient perspectives are important [55, 56]. Within the second domain of *Practices*, we found many statements that appropriate polypharmacy requires the active involvement of patients, however few discussed in detail how patients are to become involved in medication practices, instead sticking to more general assumptions or statements. Most views in this domain are represented as suggestions to increase patient empowerment, to increase patient understanding of the importance of their involvement with their medications, or regarding the need to ensure that the medication plan fits with a patient’s daily life [57, 58]. The third domain, *Values & Concerns,* examines broader aspects of polypharmacy from a structural perspective, e.g. by exploring levels of integrated care or economic models in healthcare. While this could include developing models more in favor of the healthcare economy and less regarding the needs of individual patients, it could also include a wish to develop health care systems better equipped to secure patient safety and optimal medication use from the perspective of individual users, for instance by addressing the need to include data from relevant patient populations when developing polypharmacy guidelines [59].

Finally, based on the review and analysis presented here, we argue for the importance of taking the surrounding factors of specific studies and references into consideration when analyzing different conceptualizations of inappropriate polypharmacy. The historical perspective is relevant here because polypharmacy is a relatively new phenomenon in medicine, one which has evolved dramatically over the past few decades. While the majority of references adopt the somewhat new understanding of polypharmacy as not necessarily problematic per se, we still found traces of the more outdated perception of polypharmacy as explicitly problematic [60, 61]. This serves as a proof of the ongoing heterogeneity in the understanding of this phenomenon. The amount of research on polypharmacy is obviously escalating, which makes the general discussion of not only solutions timely and relevant, but also the discussion of how the problems associated with polypharmacy are perceived differently across different settings and scientific fields.

When examining the settings of the different references included in this review, the majority were in either a primary (including long term care facilities, e.g. nursing homes) or secondary (or cross-sectional) healthcare setting, and only a few took place outside the formal healthcare system, for instance in individuals’ homes. This might be obviously due to the prescribing authority placed in clinical settings, yet most medications are administered by patients themselves or relatives at home [62, 63]. Therefore, it might be extremely relevant to focus more on inappropriate polypharmacy in the homes of patients in the future.

Likewise, the distribution of references across different scientific domains also revealed a majority of references from medical and pharmacy specialties and only a few from other health or social care-related fields. Again, the complexity of polypharmacy calls for a more nuanced description of its causes and problems in older adults, and hence could definitely benefit from perspectives outside the fields of medicine and pharmacy.

The same issues are present for studies of inappropriate polypharmacy outside Western countries. In this review, we identified few references from outside Europe, the US or Canada. While access to essential medication remains a critical problem in low- and middle income countries, there is an increase in consumption of pharmaceuticals in general in other parts of the world [1]. A more nuanced understanding of inappropriate polypharmacy could definitely be developed utilizing additional perspectives on medication use in other cultures and healthcare systems.

Finally, the majority of references included in this review discussed inappropriate polypharmacy in references representing non-original research articles. This indicates a clear need for more original research examining differences and complexities in understanding the phenomenon of inappropriate polypharmacy. Such research will lay the foundation for future implementation and improvement of current practices both for the sake of healthcare systems and the health and wellbeing of older adults.

### Strengths and limitations

The objective of this scoping review was to find and analyze different understandings of inappropriate polypharmacy presented in current research related to polypharmacy in older adults. Therefore, we did not aim for an exhaustive search, and are aware that we may have potentially missed important contributions, especially in gray literature, which constitutes a relevant limitation. However, this is in line with the scope of a scoping review. In the selection of full text readings, we aimed for a broad representation of knowledge and perspectives on polypharmacy and hence excluded studies exploring polypharmacy from the same perspective. This includes the majority of epidemiological studies using population data to extract levels of inappropriate polypharmacy that do not discuss the concepts of appropriate or inappropriate polypharmacy in any detail. In this research selection we have excluded some original research studies, which is part of the reason why with the included studies comprised more reviews and commentaries.

We were adhering to the methodological framework for Scoping reviews described by Arksey and O’Malley together with the recommendations of Levac et al [17, 18]. This is a strength and increases the internal validity of our study. Further, we followed the PRISMA extension for scoping reviews which was published in 2018 which is a checklist containing 20 reporting items and 2 optional items to include when completing a scoping review.

In the discussion, we highlighted the lack of references from outside the field of medicine. Yet we are aware that the concept of polypharmacy is primarily biomedical. Choosing alternative key words (e.g., medication use or adherence) or a search string more focused on identifying studies on patient perspectives on polypharmacy might have resulted in more studies with a patient-specific focus. However, it is important to emphasize that in the included literature, the concept of inappropriate polypharmacy is rarely found and discussed with or among patients. There seems to be a common agreement that inappropriate polypharmacy is of concern due to the potential harms caused to the individual (often older) patient, yet little research seems to explore these aspects specifically from the perspective of the users.

### Implications

This scoping review aims to contribute to a shared understanding of the concept of inappropriate polypharmacy. The framework suggested may be used to plan and appraise future studies regarding inappropriate polypharmacy.

## Conclusion

Inappropriate polypharmacy is a concept shaped by its heterogeneity. The framework suggested in this article could serve as an initial strategy for sharpening research and practice on polypharmacy in old age. Importantly, searching for a single, narrow definition of inappropriate polypharmacy may be problematic. While inappropriate polypharmacy is often used as an overall concept for safety issues linked to the prescription and use of specific types, preparations or combinations of medications (often in old age), the concept should continue to accommodate a myriad of different experiences, practices and organizational factors. There is no sign that medication use will not continue to increase globally, and thus the concept of inappropriate polypharmacy should be flexible enough to reflect the complexity and change inherent in diverse patients, organizations and healthcare systems. Furthermore, factors beyond the health system should be explored and understood better. Inappropriate polypharmacy within communities and everyday life, including cultural, financial and sociopolitical systems, which all influence the use of pharmaceuticals in old age, needs further exploration.

## Supplementary Information

**Additional file 1.** Template used for data extraction

**Additional file 2.** Overview of included references and descriptive analysis

## Data Availability

The datasets generated and/or analysed during the current study are not publicly available since it is in Danish due to all the authors being native, Danish speakers. However, the datasets are available from the corresponding author on reasonable request. All methods were carried out in accordance with relevant guidelines and regulations.
