# Supplementary material for: Disentangling concepts of inappropriate polypharmacy in old age: a scoping review
Source: BMC Public Health. 2023 Feb 4;23:245. doi: 10.1186/s12889-023-15013-2 (PMC9899389; doi:10.1186/s12889-023-15013-2)
Supplement: Supplementary file 1 — Additional file 1. Template used for data extraction [file 12889_2023_15013_MOESM1_ESM.docx]

**Additional file 1:** *Template used for data extraction*

| Author information (author, year of publication, DOI) |
| --- |
| Title |
| Aims and objectives (copied from) Abstract: Introduction: Another place: |
| Methodology used:  Quantitative  Qualitative  Mixed-methods  Others: ________________ |
| Study population:  Patients  Relatives  Healthcare professionals (including policy makers). Who? _________________________________  Not applicable  Setting:  Hospital  Community  Nursing homes  Other _______________________________ |
| Country of origin  Scandinavia (Denmark, Norway, Sweden, Finland and Iceland)  Europe (including Russia)  USA/Canada  Australia/New Zealand  Southern and Central America  Asia  Middle East and Northern Africa  Sub-Saharan Africa |
| Main findings and implications (copied from) Discussion: Conclusion: |
| Search using the search tool in Adobe Acrobat on all text parts including the word “appropriate” (which also includes “inappropriate”) (copied from)  Introduction Method Results Discussion Conclusion Other |
| Search using the search tool in Adobe Acrobat on all text parts including the word “polypharmacy” (copied from)  Introduction Method Results Discussion Conclusion Other |
| Comments |
