# Supplementary material for: Disentangling concepts of inappropriate polypharmacy in old age: a scoping review
Source: BMC Public Health. 2023 Feb 4;23:245. doi: 10.1186/s12889-023-15013-2 (PMC9899389; doi:10.1186/s12889-023-15013-2)
Supplement: Supplementary file 2 — Additional file 2. Overview of included references and descriptive analysis [file 12889_2023_15013_MOESM2_ESM.docx]

**Additional file 2***: Overview of included references and descriptive analysis*

| Included references: | Year of  publi-  cation | Setting | Scientific field | Collected scientific fields | Geographical place | Methodology |
| --- | --- | --- | --- | --- | --- | --- |
| **Advinha, A.M., et al., Medication regimen complexity in institutionalized elderly people in an aging society. International Journal of Clinical Pharmacy, 2014. 36(4): p. 750-756.** | 2014 | Nursing Homes | Clinical pharmacy | Other healthcare related fields | Europe | Quantitative |
| **Aggarwal, P., S.J. Woolford, and H.P. Patel, Multi-morbidity and polypharmacy in older people: Challenges and opportunities for clinical practice. Geriatrics (Switzerland), 2020. 5(4): p. 1-11.** | 2020 | Cross-sectional | Geriatrics | Medicine | Europe | Review |
| **Ali MU, Sherifali D, Fitzpatrick-Lewis D, Kenny M, Lamarche L, Raina P, Mangin D. Interventions to address polypharmacy in older adults living with multimorbidity: Review of reviews. Can Fam Physician. 2022 Jul;68(7):e215-e226. doi: 10.46747/cfp.6807e215. PMID: 35831093.** | 2022 | Not applicable | General Practice | Medicine | USA/Canada | Review |
| **Allin S, Martin E, Rudoler D, Church Carson M, Grudniewicz A, Jopling S, Strumpf E. Comparing public policies impacting prescribing and medication management in primary care in two Canadian provinces. Health Policy. 2021 Sep;125(9):1121-1130. doi: 10.1016/j.healthpol.2021.06.002. Epub 2021 Jun 10. PMID: 34176672.** | 2021 | Primary Care | Public Health | Other healthcare related fields | USA/Canada | Review |
| **Citation: Alshanberi, A.M. Recent Updates on Risk and Management Plans Associated with Polypharmacy in Older Population. Geriatrics 2022, 7, 97. https://doi.org/10.3390/ geriatrics7050097** | 2022 | Not applicable | Geriatrics | Medicine | Sub-Saharan Africa | Commentary/editorial/discussion pape |
| **American Geriatrics Society Updated Beers Criteria for Potentially Inappropriate Medication Use in Older Adults. Journal of the American Geriatrics Society, 2012. 60(4): p. 616-631.** | 2012 | Hospital | Geriatrics | Medicine | USA/Canada | Systematic review |
| **Anthierens, S., et al., Qualitative insights into general practitioners’ views on polypharmacy. BMC family practice, 2010. 11.** | 2010 | Primary care | General practice | Medicine | Europe | Qualitative |
| **Ariza, G., et al., Managing Drug Therapy of Older Patients in Primary and Secondary Care. Developing Drug Products in an Aging Society: From Concept to Prescribing, 2016. 24: p. 629-657.** | 2016 | Cross-sectional | Pharmaceutical sciences | Other healthcare related fields | USA/Canada | Book chapter |
| **Aubert, C.E., et al., Outcome Measures for Interventions to Reduce Inappropriate Chronic Drugs: A Narrative Review. Journal of the American Geriatrics Society. 2020** | 2020 | Cross-sectional | Geriatrics | Medicine | USA/Canada | Review |
| **Avorn, J. and J.H. Gurwitz, Drug use in the nursing home. Annals of Internal Medicine, 1995. 123(3): p. 195-204.** | 1995 | Nursing Homes | Internal Medicine | Medicine | USA/Canada | Commentary/editorial/discussion paper |
| **Bakker, L., et al., A baseline assessment by healthcare professionals of Dutch pharmacotherapeutic care for the elderly with polypharmacy. European Journal of Public Health, 2017. 27(4): p. 679-686.** | 2017 | Cross-sectional | Public Health | Other healthcare related fields | Europe | Quantitative |
| **Barnett, N.L., L. Oboh, and K. Smith, Patient-centred management of polypharmacy: A process for practice. European Journal of Hospital Pharmacy, 2016. 23(2): p. 113-117.** | 2016 | Hospital | Clinical pharmacy | Other healthcare related fields | Europe | Review |
| **Barry HE, Hughes CM. An Update on Medication Use in Older Adults: a Narrative Review. Curr Epidemiol Rep. 2021;8(3):108-115. doi: 10.1007/s40471-021-00274-5. Epub 2021 Jul 20. PMID: 34306966; PMCID: PMC8294219.** | 2021 | Not applicable | Clinical epidemiology | Medicine | Europe | Review |
| **Beard, K., Drugs in the elderly--more good than harm? Expert Opin Drug Saf, 2007. 6(3): p. 229-31.** | 2007 | Hospital | Pharmaceutical sciences | Other healthcare related fields | Europe | Commentary/editorial/discussion paper |
| **Bennett, F. and R. Sofat, Polypharmacy: The whys, the so whats and the what nexts. British Journal of Hospital Medicine, 2020. 81(5).** | 2020 | Hospital | Hospital medicine | Medicine | Europe | Commentary/editorial/discussion paper |
| **Bennett, F., R. Ferner, and R. Sofat, Overprescribing and rational therapeutics: Barriers to change and opportunities to improve. British Journal of Clinical Pharmacology. 2020** | 2020 | Hospital | Clinical pharmacology | Medicine | Europe | Commentary/editorial/discussion paper |
| **Brahma, D.K., M.D. Marak, and J.B. Wahlang, Rational use of drugs and irrational drug combinations. Internet Journal of Pharmacology, 2011. 10(1): p. 6.** | 2011 | Cross-sectional | Pharmacology | Medicine | Asia | Commentary/editorial/discussion paper |
| **Brooks CF, Argyropoulos A, Matheson-Monnet CB, Kryl D. Evaluating the impact of a polypharmacy Action Learning Sets tool on healthcare practitioners' confidence, perceptions and experiences of stopping inappropriate medicines. BMC Med Educ. 2022 Jun 27;22(1):499. doi: 10.1186/s12909-022-03556-8. PMID: 35761284; PMCID: PMC9235240.** | 2022 | Primary care | Public Health | Other healthrelated fields | Europe | Mixed-method |
| **Brunet, N.M., et al., A patient-centered prescription model assessing the appropriateness of chronic drug therapy in older patients at the end of life. European Geriatric Medicine, 2015. 6(6): p. 565-569.** | 2015 | Hospital | Geriatrics | Medicine | Europe | Qualitative |
| **Christensen, L.D., et al., Physicians' Non-Uniform Approach to Prescribing Drugs to Older Patients – A Qualitative Study. Basic and Clinical Pharmacology and Toxicology, 2017. 121(6): p. 505-511.** | 2017 | Cross-sectional | Pharmacology | Medicine | Scandinavia | Qualitative |
| **Frini, A., C. Sirois, and M.L. Laroche, A linguistic multi-criteria classification approach for the evaluation of polypharmacy quality. Lecture Notes in Engineering and Computer Science, 2018. 2.** | 2018 | Cross-sectional | Engeneering and computer sciences | Other | USA/Canada | Quantitative |
| **Gabauer, J., Mitigating the Dangers of Polypharmacy in Community-Dwelling Older Adults. AJN American Journal of Nursing, 2020. 120(2): p. 35-43.** | 2020 | Primary care | Nursing | Other healthcare related fields | USA/Canada | Commentary/editorial/discussion paper |
| **Gloth, F.M., Inappropriate prescribing: Beers criteria, polypharmacy, and drug burden. Medication Management in Older Adults: A Concise Guide for Clinicians, 2010: p. 119-125.** | 2010 | Hospital | Geriatrics/clinicians | Medicine | USA/Canada | Book chapter |
| **Gnjidic, D., et al., Polypharmacy cutoff and outcomes: Five or more medicines were used to identify community-dwelling older men at risk of different adverse outcomes. Journal of Clinical Epidemiology, 2012. 65(9): p. 989-995.** | 2012 | Primary care | Clinical epidemiology | Medicine | Australia/New Zealand | Quantitative |
| **Hajjar, E.R., A.C. Cafiero, and J.T. Hanlon, Polypharmacy in elderly patients. American Journal Geriatric Pharmacotherapy, 2007. 5(4): p. 345-351.** | 2007 | Non-applicable | Geriatric pharmacotherapy | Medicine | USA/Canada | Review |
| **Hassan, N.B., et al., Development and validation of a new Prescription Quality Index. British Journal of Clinical Pharmacology, 2010. 70(4): p. 500-513.** | 2010 | Non-applicable | Pharmacology | Medicine | Asia | Quantitative |
| **Holbeach, E. and P. Yates, Prescribing in the elderly. Australian Family Physician, 2010. 39(10): p. 728-+.** | 2010 | Primary care | General practice | Medicine | Australia/New Zealand | Commentary/editorial/discussion paper |
| **Holmes, H.M., Rational Prescribing for Patients With a Reduced Life Expectancy. Clinical Pharmacology & Therapeutics, 2009. 85(1): p. 103-107.** | 2009 | Primary care | Pharmacology | Medicine | USA/Canada | Commentary/editorial/discussion paper |
| **Holmqvist, M., et al., Older persons’ experiences regarding evaluation of their medication treatment—An interview study in Sweden. Health Expectations, 2019. 22(6): p. 1294-1303.** | 2019 | Primary care | Public Health | Other healthcare related fields | Scandinavia | Qualitative |
| **Holt, S., S. Schmiedl, and P.A. Thurmann, Potentially inappropriate medications in the elderly: The PRISCUS list. [German, English]. Deutsches Arzteblatt, 2010. 107(31-32): p. 543-551.** | 2010 | Cross-sectional | Clinical medicine/public health | Medicine | Europe | Qualitative |
| **Hughes, C.M., et al., Does organisational culture influence prescribing in care homes for older people? A new direction for research. Drugs & aging, 2007. 24(2): p. 81-93.** | 2007 | Nursing Homes | Cross-disciplinary | Other healthcare related fields | USA/Canada | Commentary/editorial/discussion paper |
| **Huisman, B.A.A., et al., Role of nurses in medication management at the end of life: A qualitative interview study. BMC Palliative Care, 2020. 19(1).** | 2002 | Cross-sectional | Palliative care | Medicine | Europe | Qualitative |
| **Kaufman G (2011) Polypharmacy in older adults. Nursing Standard. 25, 38, 49-55. Date of acceptance: April 5 2011** | 2011 | Non-applicable | Nursing | Other healthcare related fields | Europe | Commentary/editorial/discussion paper |
| **Kaufman, G., Identifying polypharmacy in the primary care setting. Practice Nursing, 2016. 27(1): p. 28-33.** | 2016 | Primary care | Nursing | Other healthcare related fields | Europe | Commentary/editorial/discussion paper |
| **Kaufman, G., Polypharmacy perspectives in general practice environments. Practice Nursing, 2019. 30(7): p. 342-347.** | 2019 | Primary care | Nursing | Other healthcare related fields | Europe | Commentary/editorial/discussion paper |
| **Kotronoulas, G., M. Cooper, and B. Johnston, Core patient-reported outcomes (PROs) and PRO measures (PROMs) for polypharmacy medicines reviews: A sequential mixed-methods study. Patient Preference and Adherence, 2019. 13: p. 2071-2087.** | 2019 | Cross-sectional | Cross-disciplinary | Other healthcare related fields | Europe | Mixed-methods |
| **Kurczewska-Michalak M, Lewek P, Jankowska-Polańska B, Giardini A, Granata N, Maffoni M, Costa E, Midão L, Kardas P. Polypharmacy Management in the Older Adults: A Scoping Review of Available Interventions. Front Pharmacol. 2021 Nov 26;12:734045. doi: 10.3389/fphar.2021.734045. PMID: 34899294; PMCID: PMC8661120.** | 2021 | Not applicable | Phamacology | Other healthcare related fields | Europe | Review |
| **Lavan, A., P. Gallagher, and D. O'Mahony, Future perspectives in drug therapy of older adults. AAPS Advances in the Pharmaceutical Sciences Series, 2016. 26: p. 737-757.** | 2016 | Non-applicable | Pharmaceutical sciences | Other healthcare related fields | Europe | Book chapter |
| **Lee GB, Etherton-Beer C, Hosking SM, Pasco JA, Page AT. The patterns and implications of potentially suboptimal medicine regimens among older adults: a narrative review. Ther Adv Drug Saf. 2022 Jul 4;13:20420986221100117. doi: 10.1177/20420986221100117. PMID: 35814333; PMCID: PMC9260603.** | 2022 | Not applicable | Pharmaceutical science | Other healthcare related fields | Australia | Review |
| **Lo SY, Reeve E, Page AT, Zaidi STR, Hilmer SN, Etherton-Beer C, McLachlan A, Pont L, Naganathan V. Attitudes to Drug Use in Residential Aged Care Facilities: A Cross-Sectional Survey of Nurses and Care Staff. Drugs Aging. 2021 Aug;38(8):697-711. doi: 10.1007/s40266-021-00874-2. Epub 2021 Jun 25. PMID: 34169458.** | 2021 | Primary Care | Geriatric | Medicine | Australia | Qualitative |
| **Lun P, Law F, Ho E, et al. Optimising prescribing practices in older adults with multimorbidity: a scoping review of guidelines. BMJ Open 2021;11:e049072. doi:10.1136/ bmjopen-2021-049072** | 2021 | Not applicable | Geriatric | Medicine | Australia | Review |
| **Magalhaes, M.S., F.S. dos Santos, and A.M.M. Reis, Factors associated with the use of potentially inappropriate medication by elderly patients prescribed at hospital discharge. Einstein-Sao Paulo, 2020. 18.** | 2020 | Hospital | Cross-disciplinary | Other healthcare related fields | South- and Middle America | Quantitative |
| **Mair, A., M. Wilson, and T. Dreischulte, Addressing the challenge of polypharmacy. Annual Review of Pharmacology and Toxicology, 2020. 60: p. 661-681.** | 2020 | Non-applicable | Pharmacology | Medicine | Europe | Review |
| **Martyn-St James, M., et al., Evidence for the impact of interventions and medicines reconciliation on problematic polypharmacy in the UK: A rapid review of systematic reviews. British Journal of Clinical Pharmacology, 2020.** | 2020 | Non-applicable | Pharmacology | Medicine | Europe | Review |
| **Matanovic, S.M. and V. Vlahovic-Palcevski, Potentially inappropriate prescribing to the elderly: comparison of new protocol to Beers criteria with relation to hospitalizations for ADRs. European Journal of Clinical Pharmacology, 2014. 70(4): p. 483-490.** | 2014 | Hospital | Pharmacology | Medicine | Europe | Quantitative |
| **McGavock, H., Prescription-related illness - A scandalous pandemic. Journal of Evaluation in Clinical Practice, 2004. 10(4): p. 491-497.** | 2004 | Hospital | Cross-disciplinary | Other healthcare related fields | Europe | Commentary/editorial/discussion paper |
| **McIntosh, J., et al., A case study of polypharmacy management in nine European countries: Implications for change management and implementation. PLoS One, 2018. 13(4).** | 2018 | Cross-sectional | Cross-disciplinary | Other healthcare related fields | Europe | Qualitative |
| **McVeigh, D.M., Polypharmacy in the older population: recommendations for improved clinical practice. Topics in Emergency Medicine, 2001. 23(3): p. 68-75.** | 2001 | Hospital | Emergency medicine | Medicine | USA/Canada | Review |
| **Medeiros-Souza, P., et al., Diagnosis and control of polypharmacy in the elderly. Revista de Saude Publica, 2007. 41(6): p. 1049-1053.** | 2007 | Cross-sectional | Public Health | Other healthcare related fields | South- and Middle America | Commentary/editorial/discussion paper |
| **Meid, A.D., et al., Longitudinal evaluation of medication underuse in older outpatients and its association with quality of life. European Journal of Clinical Pharmacology, 2016. 72(7): p. 877-885.** | 2016 | Cross-sectional | Pharmacology | Medicine | Europe | Quantitative |
| **Moen, J., et al., 'I don't know how many of these [medicines] are necessary': A focus group study among elderly users of multiple medicines. Patient Education and Counseling, 2009. 74(2): p. 135-141.** | 2009 | Primary care | Cross-disciplinary | Other healthcare related fields | Scandinavia | Qualitative |
| **Monane, M., S. Monane, and T. Semla, Optimal medication use in elders - Key to successful aging. Western Journal of Medicine, 1997. 167(4): p. 233-237.** | 1997 | Hospital | Medicine | Medicine | USA/Canada | Commentary/editorial/discussion paper |
| **Monteiro, C., et al., Medication Evaluation in Portuguese Elderly Patients According to Beers, STOPP/START Criteria and EU(7)-PIM List - An Exploratory Study. Patient Preference and Adherence, 2020. 14: p. 795-802.** | 2020 | Cross-sectional | Cross-disciplinary | Other healthcare related fields | Europe | Quantitative |
| **Morgan, L., Problematic polypharmacy: when to stop prescribing. Nursing & Residential Care, 2018. 20(11): p. 570-574.** | 2018 | Nursing Homes | Nursing | Other healthcare related fields | Europe | Commentary/editorial/discussion paper |
| **Morin, L., et al., Adequate, questionable, and inadequate drug prescribing for older adults at the end of life: a European expert consensus. European Journal of Clinical Pharmacology, 2018. 74(10): p. 1333-1342.** | 2018 | Cross-sectional | Pharmacology | Medicine | Scandinavia | Quantitative |
| **Morin, L., et al., Choosing Wisely? Measuring the Burden of Medications in Older Adults near the End of Life: Nationwide, Longitudinal Cohort Study. American Journal of Medicine, 2017. 130(8): p. 927-936.e9.** | 2017 | Non-applicable | Medicine | Medicine | Scandinavia | Quantitative |
| **Moßhammer, D., et al., Polypharmacy—An upward trend with unpredictable effects. Deutsches Arzteblatt International, 2016. 113(38): p. 627-633.** | 2016 | Primary care | Clinical medicine/public health | Medicine | Europe | Review |
| **Murphy, T.E., et al., Assessing multiple medication use with probabilities of benefits and harms. Journal of Aging and Health, 2008. 20(6): p. 694-709.** | 2008 | Non-applicable | Cross-disciplinary | Other healthcare related fields | USA/Canada | Quantitative |
| **Muth, C., et al., Multimorbidity's research challenges and priorities from a clinical perspective: The case of 'Mr Curran'. European Journal of General Practice, 2014. 20(2): p. 139-147.** | 2014 | Cross-sectional | General practice | Medicine | International | Qualitative |
| **Naples, J.G., et al., Recent Literature on Medication Errors and Adverse Drug Events in Older Adults. Journal of the American Geriatrics Society, 2016. 64(2): p. 401-408.** | 2016 | Non-applicable | Geriatrics | Medicine | International | Review |
| **Novaes, P.H., et al., The “iatrogenic triad”: polypharmacy, drug–drug interactions, and potentially inappropriate medications in older adults. International Journal of Clinical Pharmacy, 2017. 39(4): p. 818-825.** | 2017 | Primary care | Pharmacology | Medicine | South- and Middle America | Quantitative |
| **Olusanya, A., et al., Inappropriate drug use in the elderly outpatient population in a West-African metropolitan community Int J Clin Pharmacol Ther, 2019. 57(7): p. 334-344.** | 2019 | Primary care | Pharmacology | Medicine | South Saharan Africa | Mixed-methods |
| **Omar, M.S., A.H. Ariandi, and N.M. Tohit, Practical problems of medication use in the elderly Malaysians and their beliefs and attitudes toward deprescribing of medications. Journal of Research in Pharmacy Practice, 2019. 8(3): p. 105-111.** | 2019 | Primary care | Pharmacy | Other healthcare related fields | Asia | Quantitative |
| **Ong, S.M., et al., Variation of polypharmacy in older primary care attenders occurs at prescriber level. BMC geriatrics, 2018. 18(1).** | 2018 | Primary care | Geriatrics | Medicine | Asia | Quantitative |
| **Palmer, K., et al., Association of polypharmacy and hyperpolypharmacy with frailty states: a systematic review and meta-analysis. European Geriatric Medicine, 2019. 10(1): p. 9-36.** | 2019 | Cross-sectional | Geriatrics | Medicine | Europe | Systematic review |
| **Parsons, C., Polypharmacy and inappropriate medication use in patients with dementia: an under-researched problem. Therapeutic Advances in Drug Safety, 2017. 8(1): p. 31-46.** | 2017 | Cross-sectional | Pharmaceutical sciences | Other healthcare related fields | Europe | Review |
| **Pazan, F., J. Kather, and M. Wehling, A systematic review and novel classification of listing tools to improve medication in older people. European Journal of Clinical Pharmacology, 2019. 75(5): p. 619-625.** | 2019 | Non-applicable | Pharmacology | Medicine | Europe | Systematic review |
| **Pazan F, Wehling M. Polypharmacy in older adults: a narrative review of definitions, epidemiology and consequences. Eur Geriatr Med. 2021 Jun;12(3):443-452. doi: 10.1007/s41999-021-00479-3. Epub 2021 Mar 10. PMID: 33694123; PMCID: PMC8149355.** | 2021 | Not applicable | Geriatric | Medicine | Europe | Review |
| **Pereira, F., et al., Polypharmacy among home-dwelling older adults: The urgent need for an evidence-based medication management model. Patient Preference and Adherence, 2019. 13: p. 2137-2143.** | 2019 | Primary care | Cross-disciplinary | Other healthcare related fields | Europe | Commentary/editorial/discussion paper |
| **Petrovic M, O'Mahony D, Cherubini A. Inappropriate prescribing: hazards and solutions. Age Ageing. 2022 Feb 2;51(2):afab269. doi: 10.1093/ageing/afab269. PMID: 35136899.** | 2022 | Not applicable | Geriatric | Medicine | Europe | Commentary/editorial/discussion pape |
| **Pohontsch, N.J., et al., General practitioners' views on ( long- term) prescription and use of problematic and potentially inappropriate medication for oldest- old patients- A qualitative interview study with GPs ( CIM- TRIAD study). BMC family practice, 2017. 18.** | 2017 | Non-applicable | General practice | Medicine | Europe | Qualitative |
| **Rankin A, Cadogan CA, Barry HE, Gardner E, Agus A, Molloy GJ, Gorman A, Ryan C, Leathem C, Maxwell M, Gormley GJ, Ferrett A, McCarthy P, Fahey T, Hughes CM; PolyPrime team. An external pilot cluster randomised controlled trial of a theory-based intervention to improve appropriate polypharmacy in older people in primary care (PolyPrime): study protocol. Pilot Feasibility Stud. 2021 Mar 19;7(1):77. doi: 10.1186/s40814-021-00822-2. PMID: 33741071; PMCID: PMC7977311.** | 2021 | Primary Care | General practice | Medicine | Europe | Quantitative |
| **Reeve, J., Avoiding harm: Tackling problematic polypharmacy through strengthening expert generalist practice. British Journal of Clinical Pharmacology, 2020.** | 2020 | Non-applicable | Pharmacology | Medicine | Europe | Commentary/editorial/discussion paper |
| **Rocchiccioli, J.T., J. Sanford, and B. Caplinger, Polymedicine and aging: Enhancing older adult care through advanced practitioners. Journal of Gerontological Nursing, 2007. 33(7): p. 19-24.** | 2007 | Hospital | Nursing | Other healthcare related fields | USA/Canada | Commentary/editorial/discussion paper |
| **Ross, A. and J. Gillett, Confronting Medicine’s Dichotomies: Older Adults’ Use of Interpretative Repertoires in Negotiating the Paradoxes of Polypharmacy and Deprescribing. Qualitative health research, 2020. 30(3): p. 448-457.** | 2020 | Non-applicable | Qualitative health research | Other healthcare related fields | USA/Canada | Qualitative |
| **Salam, A., et al., Polypharmacy: Cure or curse? Quality in Ageing, 2008. 9(1): p. 24-28.** | 2008 | Hospital | Cross-disciplinary | Other healthcare related fields | Europe | Commentary/editorial/discussion paper |
| **Schöpf, A.C., et al., Elderly patients' and GPs' perspectives of patient–GP communication concerning polypharmacy: a qualitative interview study. Primary Health Care Research & Development (Cambridge University Press / UK), 2018. 19(4): p. 355-364.** | 2018 | Non-applicable | Primary health care | Other healthcare related fields | Europe | Qualitative |
| **Sharp, C.N., M.W. Linder, and R. Valdes, Jr., Polypharmacy: a healthcare conundrum with a pharmacogenetic solution. Crit Rev Clin Lab Sci, 2019: p. 1-20.** | 2019 | Non-applicable | Clinical laboratory sciences | Medicine | USA/Canada | Review |
| **Sirois, C., et al., Benefits, risks and impacts on quality of life of medications used in multimorbid older adults: a Delphi study. International Journal of Clinical Pharmacy, 2020. 42(1): p. 40-50.** | 2020 | Non-applicable | Pharmacology | Medicine | Europe | Qualitative |
| **Sirois, C., et al., Mixed Bag "Polypharmacy": Methodological Pitfalls and Challenges of This Exposure Definition. Current Epidemiology Reports, 2019. 6(3): p. 390-401.** | 2019 | Non-applicable | Epidemiology | Medicine | Europe | Review |
| **Sirois, C., et al., Polypharmacy in multimorbid older adults: Protocol for a systematic review. Systematic Reviews, 2017. 6(1).** | 2017 | Cross-sectional | Systematic reviews | Other | USA/Canada | Systematic review |
| **Sperling, L., Polypharmacy and the senior citizen: A qualitative study on the influence of direct-to-consumer advertising. 2016. 76.** | 2016 | Nursing Homes | Dissertation abstract journal | Other | USA/Canada | Qualitative |
| **Stegemann, S., et al., Geriatric drug therapy: Neglecting the inevitable majority. Ageing Research Reviews, 2010. 9(4): p. 384-398.** | 2010 | Non-applicable | Cross-disciplinary | Other healthcare related fields | Europe | Review |
| **Steinman, M.A. and J.T. Hanlon, Managing medications in clinically complex elders: "There's got to be a happy medium". JAMA - Journal of the American Medical Association, 2010. 304(14): p. 1592-1601.** | 2010 | Non-applicable | Medicine | Medicine | USA/Canada | Review |
| **Stevenson, J.M., J.G. Davies, and F.C. Martin, Medication-related harm: a geriatric syndrome. Age and Ageing, 2020. 49(1): p. 7-11.** | 2020 | Non-applicable | Cross-disciplinary | Other healthcare related fields | Europe | Commentary/editorial/discussion paper |
| **Stuijt, C.C.M., et al., Reliability of the medication appropriateness index in Dutch residential home. Pharmacy World & Science, 2009. 31(3): p. 380-386.** | 2009 | Nursing Homes | Pharmacy | Other healthcare related fields | Europe | Quantitative |
| **Taghy, N., et al., Failure to reach a consensus in polypharmacy definition: An obstacle to measuring risks and impacts—results of a literature review. Therapeutics and Clinical Risk Management, 2020. 16: p. 57-73.** | 2020 | Non-applicable | Clinical therapeutics | Medicine | Europe | Review |
| **Taghy N, Cambon L, Boulliat C, Aromatario O, Dussart C. Exploring the Determinants of Polypharmacy Prescribing and Dispensing Behaviours in Primary Care for the Elderly-Protocol for a Qualitative Study. Int J Environ Res Public Health. 2021 Jul 19;18(14):7656. doi: 10.3390/ijerph18147656. PMID: 34300106; PMCID: PMC8303935.** | 2021 | Primary Care | Public Health | Other healthcare related fields | Europe | Qualitative |
| **Tasaka, Y., et al., Potential drug-related problems detected by routine pharmaceutical interventions: safety and economic contributions made by hospital pharmacists in Japan. Journal of Pharmaceutical Health Care and Sciences, 2018. 4.** | 2018 | Hospital | Pharmaceutical sciences | Other healthcare related fields | Asia | Quantitative |
| **Tegegn, H.G., et al., Medication-related quality of life among Ethiopian elderly patients with polypharmacy: A cross-sectional study in an Ethiopia university hospital. PLoS One, 2019. 14(3).** | 2019 | Hospital | Cross-disciplinary | Other healthcare related fields | Northern Africa | Quantitative |
| **Thiem, U., et al., Prerequisites for a new health care model for elderly people with multimorbidity The PRISCUS research consortium. Zeitschrift fur Gerontologie und Geriatrie, 2011. 44(2): p. 115-120.** | 2011 | Non-applicable | Geriatrics | Medicine | Europe | Commentary/editorial/discussion paper |
| **Thomas, R.E. and B.C. Thomas, A systematic review of studies of the stopp/start 2015 and american geriatric society beers 2015 criteria in patients >= 65 years. Current Aging Science, 2019. 12(2): p. 121-154.** | 2019 | Hospital | Cross-disciplinary | Other healthcare related fields | Europe | Review |
| **Todd A, Holmes HM. Recommendations to support deprescribing medications late in life. Int J Clin Pharm. 2015 Oct;37(5):678-81. doi: 10.1007/s11096-015-0148-6. PMID: 26078120; PMCID: PMC4682203** | 2015 | Non-applicable | Pharmacology | Medicine | USA/Canada | Commentary/editorial/discussion paper |
| **Todd, A., et al., Inappropriate prescribing of preventative medication in patients with life-limiting illness: a systematic review. Bmj Supportive & Palliative Care, 2017. 7(2): p. 113-121.** | 2017 | Cross-sectional | Palliative care | Medicine | Europe | Systematic review |
| **Tseng, H.M., et al., Developing a measure of medication-related quality of life for people with polypharmacy. Quality of Life Research, 2016. 25(5): p. 1295-1302.** | 2016 | Hospital | Cross-disciplinary | Other healthcare related fields | Asia | Quantitative |
| **Tully, M.P., Appropriate prescribing. Reviews in Clinical Gerontology, 1996: p. 49-56.** | 1996 | Non-applicable | Geriatrics | Medicine | Europe | Review |
| **Turner, J.P., et al., Polypharmacy cut-points in older people with cancer: how many medications are too many? Supportive Care in Cancer, 2016. 24(4): p. 1831-1840.** | 2016 | Hospital | Cancer care | Medicine | Australia/New Zealand | Systematic review |
| **Varghese, D., C. Ishida, and H. Haseer Koya, Polypharmacy. StatPearls, 2020.** | 2020 | Non-applicable | Medicine | Medicine | USA/Canada | Book chapter |
| **Vass, M. and C. Hendriksen, Medication for older people--aspects of rational therapy from the general practitioner's point of view. Z Gerontol Geriatr, 2005. 38(3): p. 190-5.** | 2005 | Non-applicable | Geriatrics | Medicine | Scandinavia | Commentary/editorial/discussion paper |
| **Vass, M. and C. Hendriksen, Polypharmacy and older people--the GP perspective. Z Gerontol Geriatr, 2005. 38 Suppl 1: p. I14-7.** | 2005 | Primary care | Geriatrics | Medicine | Scandinavia | Commentary/editorial/discussion paper |
| **Veehof, L.J.G., B. Meyboom-De Jong, and F.M. Haaijer-Ruskamp, Polypharmacy in the elderly - A literature review. European Journal of General Practice, 2000. 6(3): p. 98-106.** | 2000 | Nursing Homes | General practice | Medicine | USA/Canada | Review |
| **Waller, S., Reducing medication‐related harm through Quality Use of Medicines. Journal of Pharmacy Practice & Research, 2020. 50(5): p. 374-376.** | 2020 | Non-applicable | Pharmacy | Other healthcare related fields | Australia/New Zealand | Commentary/editorial/discussion paper |
| **Wasserfallen, J.B., et al., Composition and cost of drugs stored at home by elderly patients. Annals of Pharmacotherapy, 2003. 37(5): p. 731-737.** | 2003 | Hospital | Pharmacotherapy | Medicine | Europe | Systematic review |
| **Yang, J., et al., Drug-related problems among community-dwelling older adults in mainland China. International Journal of Clinical Pharmacy, 2018. 40(2): p. 368-375.** | 2018 | Non-applicable | Pharmacy | Other healthcare related fields | Asia | Quantitative |
| **Yong, T.Y. and K.S.F. Khow, Prescribing appropriately in frail older people. Healthy Aging Research, 2015. 4.** | 2015 | Non-applicable | Cross-disciplinary | Other healthcare related fields | Australia/New Zealand | Review |
